# Establishing an Ehlers-Danlos Syndrome Clinic: Lessons Learned

**DOI:** 10.1007/s42399-022-01218-w

**Published:** 2022-07-05

**Authors:** Dacre R. T. Knight, Sunnie M. Confiado, Katelyn A. Bruno, DeLisa Fairweather, Andrea M. Seymour-Sonnier, Angita Jain, Jessica M. Gehin, Emily R. Whelan, Joshua H. Culberson, Bala Munipalli, Nancy L. Dawson, Todd D. Rozen, Joseph J. Wick, Archana Kotha

**Affiliations:** 1 Department of General Internal Medicine, Mayo Clinic, 4500 San Pablo Rd, Jacksonville, FL 32224 USA; 2 Department of Clinical Genomics, Mayo Clinic, Jacksonville, FL USA; 3 Department of Cardiovascular Medicine, Mayo Clinic, Jacksonville, FL USA; 4 Department of Neurology, Mayo Clinic, Jacksonville, FL USA; 5 Department of Research Services, Mayo Clinic, Rochester, MN USA

**Keywords:** Ehlers-Danlos syndrome, Hypermobility spectrum disorder, Hypermobile Ehlers-Danlos syndrome, Patient focus group, Translational research

## Abstract

In a large academic medical center, patient requests from the community and internal referrals for evaluation of suspected hypermobility conditions were being denied consultation because services specific to this condition were not available. We identified this gap and developed a comprehensive evaluation for this unique patient population. The objective of this paper is to demonstrate a solution for improving outcomes in a neglected patient population by establishing an innovative outpatient clinic specifically tailored for patients with EDS.

We describe the lessons learned on establishing a specialty clinic for treating patients with hypermobility syndromes including hypermobile Ehlers-Danlos syndrome (**hEDS**) and hypermobile syndrome disorder (**HSD**). Findings were collected from a patient focus group that was instrumental in understanding common care gaps. We document the firsthand perspective of three patients presenting with hypermobility accompanied by joint pain and denote the complicated state of healthcare in recognizing and treating this condition. A summary of patient demographics and characteristics was collected from patients seen in the clinic from November 14, 2019 to April 13, 2021.

The firsthand accounts illustrate the challenges faced in treating this condition and the need for, and success of, this clinic using a coordinated care model. Demographics reveal a primarily white female population under the age of 50 with many comorbidities. Genetic testing was largely negative, with more patients diagnosed with HSD than hEDS.

Our shared experience of launching a successful EDS clinic may assist other clinicians in establishing similar care models.

## Introduction

Ehlers-Danlos syndrome (**EDS**) is a heterogeneous group of hereditary connective tissue disorders, characterized by joint hypermobility, skin hyperextensibility, easy bruising and tissue fragility [1–3]. These features are present in all EDS subtypes in varying degrees and help to differentiate EDS from other hypermobility disorders. EDS affects approximately one in 5000 people worldwide [3–6]. The genetic (i.e., molecular) cause of EDS is known for all but one named subtype. Variants in 20 different genes encode fibrillary collagen types I, III and V, encode modifying and processing enzymes for these proteins, and/or encode enzymes that modify glycosaminoglycan chains of proteoglycans that lead to connective tissue dysfunction [2, 7]. Because EDS can involve essentially every organ system, it is a challenging disorder to diagnose and manage. EDS features overlap with other connective tissue disorders including Marfan syndrome and osteogenesis imperfecta [3, 8–11].

No identifiable gene has been associated with hypermobile EDS (**hEDS**) or hypermobility syndrome disorder (**HSD**). Patients with hEDS and HSD are diagnosed based on clinical criteria developed by the International Consortium on EDS that was updated in 2017 [3, 12, 13]. EDS was once considered relatively rare [3, 14]. Although increased awareness in the medical community and increased patient awareness through online access have resulted in greater identification of this disorder in recent years, there remains a large area of unmet needs for patients suffering from EDS [15]. Because of variations in symptom presentation, symptom severity, and lack of a genetic marker, diagnosis has been estimated to be delayed up to 14 years [16, 17]. For 25% of patients, it took over 28 years to obtain an hEDS diagnosis [16, 17]. For those reasons, the prevalence of hEDS/HSD is suspected to be higher than reported [18].

Despite recent advances in genetic diagnosis, testing and research, there is a significant gap in care for patients with hEDS/HSD. To date, there are no specific medical or genetic therapies available to care for patients with any type of EDS. There are other well-known specialty genetics clinics worldwide [19], but this is the first known report on establishing a dedicated EDS clinic at a large academic medical center. The EDS Clinic at Mayo Clinic in Florida was started in November 2019 to provide diagnosis and specialized care in the hope of advancing the science of these disorders. Barriers to care for hEDS/HSD patients include limited primary care education and overburdened genetic practices. To address these barriers and to provide a blueprint for clinic development, we established a de novo outpatient EDS clinic within an academic adult general internal medicine practice and monitored its progress over 1 year. Little is published documenting the benefits of caring for these patients, and even less on establishing a focused medical practice for patients with hEDS/HSD. Herein, we provide our experience with establishing an EDS clinic.

### Methods

#### Clinic Demographics

Retrospective review of the demographic and clinical data from the medical records reported in this manuscript was approved by the Mayo Clinic Institutional Review Board (IRB# 19–011,260) and informed consent was waived by the Institutional Review Board for all patients. The research conformed to the principles outlined in the Declaration of Helsinki.

#### Statistical Analysis

Continuous variables were summarized as mean (average) or range (minimum and maximum) where appropriate and categorical variables were reported as frequency (percentage). The analyses were conducted using Prism.

### Results

#### The Clinic

To lay the foundation for starting the clinic, specific personnel were required, including an administrator, a physician, a dedicated full-time nurse, a scheduler, and a medical geneticist available for referrals. The EDS Clinic calendar is consistently full, and the clinic operates four half days per week typically seeing 4 patients a day. There has been no formal advertisement of the EDS Clinic outside of our institution, meaning that patients who self-refer are made aware of the clinic primarily through internet searches (The EDS Society Directory), discussion forums, and word of mouth. The appointments are 60 min long. Once evaluated in the EDS Clinic, the physician recommends subspecialty consultations based on medical needs. Consult appointments were arranged as close to original appointment as possible, especially for patients traveling from great distances, but most subspecialty appointments were obtained within 2–3 months of entering the Clinic. The major consults that were recommended in this cohort of patients in the first year of the Clinic based on the intake questionnaire are summarized in Table 1.

**Table 1:** Recommended consults for patients seen at the EDS Clinic based on the intake questionnaire

Referral consult	% ‡
Physical Therapy	100.0
Occupational Therapy	100.0
POTS Clinic	92.4
Gastroenterology	88.6
Pain Clinic	85.2
Cardiology	85.2
Psychology	82.5
Neurology	84.8
Sleep Center	76.3
Fibromyalgia Clinic	71.4
Women’s Health	67.0
Pelvic Floor Therapy	47.6
Dermatology	33.8
Rheumatology	33.0

^‡^ %: percentage

Unique to this EDS Clinic, patient care was integrated with research from its inception to gain a better understanding of the underlying mechanisms that contribute to this understudied condition. The link between physicians and researchers/basic research was critical for promoting advances in translating knowledge to the clinical practice. Research staff working on the study were largely volunteer until enough data could be collected to obtain external funding to support research and staff effort. Research personnel financially supported by the Clinic include an Associate Professor and supplies for collecting and processing blood samples. Volunteer research staff include an Assistant Professor, Research Fellow, and three technicians/students. All patients were invited to participate in research; blood samples were obtained if consent was given.

Research on data and samples is conducted on an on-going basis. Current areas of research include, but are not limited to, sex differences, fibromyalgia, mast cell disorders, telemedicine, genetic pathology, and population studies. Research findings are published and shared on our Clinic website (https://connect.mayoclinic.org/blog/ehlers-danlos-syndrome/) after publication, in which patients are invited to “follow” for research updates as they become available.

**Fig. 1:**
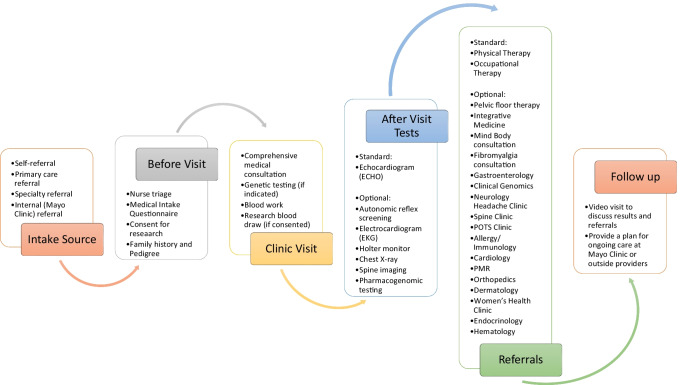
**EDS Clinic workflow.** This figure describes the intake of patients to the EDS Clinic and each step through their care

The workflow of the EDS Clinic was specifically designed to allow comprehensive assessments, testing and follow-up (Fig. 1). Patients were asked to complete a standard of care intake questionnaire online before their first appointment. At the initial visit, the patient was evaluated by gathering personal, family, and medical history, creating a genetic pedigree, performing a full physical exam, cardiac imaging, and individualized laboratory assessments. Cardiac imaging included echocardiography, evaluating for valve prolapse and aortic root dilatation based on strict echocardiographic criteria [20]. Upon this evaluation, and if indicated by concern for an EDS subtype other than hEDS, genetic phenotype-driven panel testing was conducted by the Mayo Clinic Medical Laboratory after obtaining authorization for genetic testing from the patient. The Ehlers-Danlos syndrome 12-gene panel (ADAMTS2, ATP7A, CHST14, COL1A1, COL1A2, COL3A1, COL5A1, COL5A2, FKBP14, FLNA, PLOD1, and SLC39A13) was used. All genes identified as known causes of EDS are included in this panel. Separate broader connective tissue panels were ordered if there was concern for a separate non-EDS inherited connective tissue disorder. A prior authorization process for genetic testing coverage was performed independently between Mayo Clinic and the patient’s insurance. Of the 483 patients who had genetic testing results from November 2019 through April 2021, 73 patients provided previous results and 410 patients completed genetic testing through the EDS Clinic. Of the 410 patients, 255 patients utilized the commercial lab.

Pathogenicity of genetic variants were determined by the designated lab after review of known variants, genetic conservation, and prediction of changes to protein structure. In patients where novel variants were identified and classified as variants of uncertain significance (**VUS**), the EDS Clinic coordinated a referral to clinical genomics.

A second visit was conducted by video to review lab results, imaging and, if ordered — genetic testing results. They also received education about their condition. Referrals for additional specialty appointments and diagnostic testing were placed based on the individual medical needs.

Patients returned to Mayo Clinic to complete specialty appointments and diagnostic testing. At that time, patients had the opportunity to develop treatment plans with individual specialties. This multidisciplinary team approach ensured patients were evaluated and treated using a wide array of modalities and treatment plans. Shared decision-making between provider and patient directed the course of treatment.

The final visit was held by telehealth. This appointment served to summarize and integrate all previous referrals and diagnostic testing and outline treatment recommendations for continued care with the primary care provider, which was often not at our institution. There are cases where primary care physicians lack sufficient expertise or interest in caring for patients with hEDS/HSD. However, every patient maintains direct access to the EDS Clinic care team for significant medical issues that arise following their evaluations. Furthermore, every patient is given an individual care plan and documentation to share with their primary care team.

#### Patient and Advisory Focus Groups

When the EDS Clinic was first being designed, Mayo Clinic in Florida invited a cohort of hEDS and HSD patients (*n* = 6) to a targeted focus group session. The objective was to receive and utilize feedback from a representative group that included patients from diverse demographic backgrounds. Patients identified several major themes, including obtaining the right care at the right time, access to multidisciplinary, holistic, and coordinated care and additional educational resources. The Mayo Clinic EDS Clinic continues to meet with EDS/HSD patient focus groups to gain perspective on ongoing patient concerns.

The Clinic members also meet monthly with an EDS Advisory Forum consisting of clinical and research stakeholders with the goal of providing education, encouraging, and disseminating research and improving patient care.

#### Patient Perspectives

Many of the patients seen over the year are not only aware of current EDS research but are also cognizant of deficiencies in the patient care community for people with hEDS/HSD. We share the viewpoint of three of our hEDS/HSD advisory group patients with their consent.

*“Through the years I have seen a need for centers of excellence for EDS. The hope is that that there will be multiple people working together while sharing knowledge and research.*

*As an EDS patient and advocate, I view the EDS Clinic as a resource for local doctors but also for me and for those I support as a place to go to get answers and help. Ultimately, I would like my grandchildren and nieces to avoid suffering my fate.*” **[*KA*].**

*“From an early age I had what my orthopedist told me were growing pains. Because I was taller than most of my classmates, I did my best to accept his answer, although I always wondered if it weren't something more (especially after I stopped growing!).*

*Now I'm devouring any information I can get on hEDS to manage it and get as optimal health as possible.”* **[*EE*].**

*“After 20* + *years of receiving wrong diagnoses (conversion disorder, psychological pain, *etc*.) and after having had countless visits and exams in Italy and the US, I decided to take the matter in my hands. Around that time, I tore my ACL and fractured the tibial plateau just by standing up and the doctors could not explain it. I started studying genetic diseases connected to some of my symptoms and when I found out about the existence of EDS, I knew it was what I was suffering from.*

*My experience at the EDS clinic was life changing. I was so blown-away at the preparation, brilliancy and intelligence, communication skills and genuine care for the patient of the clinic providers that in just a few weeks I made major changes to my life—activities, priorities, scheduling, *etc*. – as they had suggested (something that I would have never thought possible!). What strikes me the most is the doctors’ ability to see things in an integrated way, and care for my wellness more than for my health.”* **[MS].**

#### Clinic Demographics

Between November 14, 2019 and April 13, 2021, 563 adult (≥ 18 years of age) patients entered the Mayo Clinic Florida EDS Clinic. Any adult patient with suspected joint hypermobility or a possible connective tissue disorder was scheduled. Most patients were female (91.9%) with only 44 males (7.8%) and two non-binary patients.

Of the 563 patients who were seen at the EDS Clinic during this time, 503 patients completed the intake questionnaire and demographic comparisons are made with this subpopulation. In this subgroup, of the 92.0% of patients that were female (age range: 18–75), 86.1% were under 50 years of age. Of the 7.6% of patients that were male (age range: 18–60), 94.7% were under 50 years of age. Most patients were White (93.0%), with few Hispanic or Latino patients (6.7%), and patients had varying levels of education (Table 2). In the intake questionnaire, patients were asked if they were previously assessed for some form of hypermobility. Of the 503 patients who answered this question, 55.0% stated they had been assessed previously, with 52.5% diagnosed with hEDS and 9.9% with HSD. Only 7.6% of the patients had multiple family members seen at the Clinic, but many patients reported that multiple members of their family were also hypermobile (60.0%) (Table 3).

**Table 2:** Patient demographics during the first year of the EDS Clinic

		*n*†	% ‡
**Sex (***n* = 503)			
	*Females*	*463*	*92.0*
	Females ≤ 50 at diagnosis	399	86.1
	Females > 50 at diagnosis	64	13.9
	*Males*	*38*	*7.6*
	Males ≤ 50 at diagnosis	36	94.7
	Males > 50 at diagnosis	2	5.3
	*Non-Binary (NB)*	*1*	*0.2*
	NB ≤ 50 at diagnosis	*1*	100
	NB > 50 at diagnosis	0	0
	*Choose not to disclose/Unknown*	*1*	*0.2*
	Mean age at diagnosis Females (Range: 18–75) Males (Range: 18–60)	34.6 26.8	
Race (*n* = 503)	Patients were able to choose multiple answers		
	American Indian/Alaska Native	3	0.5
	Asian	4	0.7
	African American	2	0.3
	Native Hawaii/ Pacific Islander	0	0
	White	468	93.0
	Multiple race	15	2.9
	Other	6	1.1
	Unknown	8	1.5
Ethnicity (*n* = 503)			
	Hispanic/Latino	33	6.7
	Not Hispanic Latino	451	89.6
	Unknown	19	3.7
Highest level of education (*n* = 503)			
	Some high school, no diploma	6	1.2
	High school graduate, diploma or GED	30	6.0
	Some college	124	24.6
	Trade/technical/vocational school	20	4.0
	Associate degree	57	11.3
	Bachelor’s degree	147	29.2
	Master’s degree	70	14.0
	Professional/Doctorate (JD, MD, PhD, etc.) degree	19	3.7
	Choose not to disclose	2	0.4
	Unknown	28	5.6
Residence (*n* = 503)			
	Florida	323	64.2
	Georgia	61	12.1
	Alabama	16	3.2
	North Carolina	17	3.4
	South Carolina	23	4.6
	Other states	61	12.1
	Unknown	2	0.4

^†^ n: number

^‡^ %: percentage

**Table 3:** History of hypermobility

		*n*†	% ‡
Assessed previously for hypermobility (*n* = 503)			
	Yes	277	55.0
	No	200	39.8
	Unknown	22	4.4
	Unanswered	4	0.8
Previously diagnosed with the following conditions (*n* = 303)	Patients were able to choose multiple answers		
	Hypermobile Syndrome	59	19.5
	Hypermobile Spectrum Disorder	30	9.9
	Hypermobile Ehlers-Danlos Syndrome	159	52.5
	General Ehlers-Danlos Syndrome	28	9.2
	Other types of EDS§	12	4.0
	No previous diagnosis	22	7.3
	Unknown/ Not answered	16	5.3
Previous genetic testing (*n* = 277)			
	Yes	94	33.9
	No	172	62.1
	Unknown	12	4.0
	Unanswered	226	
Family member with similar diagnosis (*n* = 503)			
	Yes	302	60.0
	No	132	26.2
	Unknown	59	11.8
	Not answered	10	2.0
Severity of family’s symptoms in relation to your symptoms (*n* = 302)	Patients were able to choose multiple answers		
	Less severe	141	46.7
	Same	87	28.8
	More severe	70	23.1
	Unknown	48	15.9
	Unanswered	157	52.0
Have family members been seen in the EDS Clinic? (*n* = 503)			
	Yes	38	7.6
	No	260	51.8
	Unanswered	204	40.6

^†^ n: number

^‡^ %: percentage

^§^ EDS: Ehlers-Danlos syndrome

Patients who had not received genetic testing previously were offered genetic testing as part of the EDS Clinic. The average cost to the patient who underwent genetic testing was $343, with a range from $0 to $600 and results were obtained in 45 days on average, ranging from 15 to 60 days. Of the patients seen through April 13, 2021 (*n* = 563), 76 patients did not have genetic testing completed, four patients had pending genetic testing results and 483 had received genetic testing results. Of the 483 patients with results, 338 had no findings of pathogenic connective tissue variants (70%), 136 had a VUS or an inconclusive result (28.1%) and nine patients had pathogenic or likely pathogenic variants (1.9%) (Table 4).

**Table 4:** Genetic testing results for EDS Clinic patients

	(Total = 483) *n*†	% ‡
No pathogenic connective tissue variants	338	70.0
Variant of unknown significance or inconclusive result	136	28.1
Pathogenic variants/ variant of unknown likely pathogenic	9	1.9

^†^ n: number

^‡^ %: percentage

Most patients seen at the EDS Clinic were diagnosed either with hEDS or HSD. Physician diagnosis was extracted for 489 patients revealing that 40.5% were diagnosed with hEDS, 47.6% were diagnosed with HSD, and 11.9% were not diagnosed with a hypermobile connective tissue condition (Table 5).

**Table 5:** Documented diagnosis after physician’s evaluation at the EDS Clinic

	(Total = 563) *n*†	% ‡
hEDS§	221	39.2
HSD¶	269	47.8
Does not meet criteria for hEDS/HSD	73	13.0

^†^ n: number

^‡^ %: percentage

^§^ hEDS: hypermobile Ehlers-Danlos syndrome

^¶^ HSD: hypermobile spectrum disorder

The most common self-reported complaints (comorbidities) are included in Table 6.

**Table 6:** Common comorbidities self-reported by patients seen at the EDS Clinic

Common comorbidities/diagnosis	(Total = 503) *n*†	% ‡
Chronic pain (pain lasting > 3–6 months)	383	76.1
Brain Fog	382	75.9
Headache	352	70.0
Anxiety	337	67.0
Chronic fatigue	303	60.2
Constipation	300	59.6
Nausea	297	59.0
Depression	278	55.3
Migraine	261	51.9
Diarrhea	258	51.2
Tinnitus	249	49.5
Cervicalgia	226	45.0
Insomnia	213	42.3
Vertigo	209	41.5
Sleep disturbances	204	40.6
Autonomic dysfunction	180	35.7
Fibromyalgia	156	31.0

^†^ n: number

^‡^ %: percentage

### Lessons Learned

Continuous improvement based on patient feedback is a foundational element of the Clinic. Patient expectations were closely monitored during the first year and continue to be evaluated to enable improvement. As this is a novel report, we are eager to share this experience as soon as possible. Below are the major topics the team focused on in the first year of the new clinic.

#### Genetic Testing

From our experience, and as described by Sulli et al. on unmet needs — lack of practice guidelines, we recognize a wide variation in protocol among providers treating patients for EDS [15]. Testing is based on clinical findings, as this is the role of the physician. Clinical findings focus on connective tissue exams and family history. Among the majority we observed, patients more readily entered the path to functional restoration upon gaining their own acceptance of a diagnostic test result. Clinician reassurance alone in our experience was not adequate for a small number of patients interested in ruling out more severe subtypes of EDS. As patient buy-in is pivotal to good outcomes, we recognize the importance of further research on outcomes to develop the best evidence-based process for genetic testing. Strictly speaking, a diagnosis of hEDS/HSD does not warrant genetic testing as there is currently no positive result nor change in management to be found for these diagnoses. Others have made good points against haphazard genetic testing [21]. However, overlap with similar conditions for which genetic testing is indicated does confuse the matter, and largely relies on clinical expertise to evaluate the medical history and physical examination to determine the utility of a genetic test. The more proficient and experienced clinician will be able to target a smaller range of connective tissue diseases to a single panel. A rich debate is to be had whether the safer, if not costlier approach is to order broad panels, or even exome studies. The cost of exome sequencing is rapidly falling. For now, it must be left to the clinician’s designation and pretest probability assessment for whichever disorder may be considered beyond the non-testable hEDS/HSD.

#### Pre-Visit Planning

The patient journey was designed in a series of three distinct stages that included (1) obtaining the history, pedigree and completing testing, (2) reviewing testing results and outlining the plan of care for future comprehensive evaluation, and (3) completion of the multispecialty comprehensive evaluation of medical concerns. To prevent delays in overall care, the clinical team began pre-scheduling specialty consults based on the results obtained in the intake questionnaires. When the patient was examined, appointments were cancelled or added based on individual medical needs.

#### Electronic Communication

The level of clerical burden associated with electronic communication between the care team and the patients was not anticipated to the extent observed with this population. The clinical team created an orientation letter that briefed patients on the clinic, outlined what to expect and answered frequently asked questions. The information helped redirect the focus on clinical questions versus operational or general questions. Electronic consent for research was implemented to reduce workload to the clinical team at the initial day of visit.

#### Expertise of Clinical Team

Our full-time equivalent structure of the core team is as follows: physician (general internal medicine), nurse, physical therapy, and occupational therapy for each patient. For this cohort, care was provided with at least one specialist in internal medicine, nursing, physical therapy, occupational therapy, psychology, medical genomics, cardiology, neurology, and/or gastroenterology. Allied health professionals were also an invaluable part of the care process. These include a mind–body therapist, health and wellness coach, and dietician. There is not yet an accredited specialty training curriculum for EDS outside of a clinical genetics residency, so expertise is gained from research, self-education, and clinical experience. This applies to physicians, as well as nursing, and therapy staff involved in the clinic. Determination for the specialty referral was made based on a clinical evaluation of comorbid conditions. These are highlighted in Table 6 and include functional gastrointestinal disorders, headache disorders, dysautonomia, fibromyalgia and chronic pain, among others. The more versed any specialist is in EDS treatment, the better the care as the optimal treatment approach is dynamic such that treatment modalities in one discipline may interfere with another discipline. Therefore, it is beneficial to have an experienced general medical physician to balance the care where such conflict might arise.

#### Virtual Visits

Three months after the new clinic opened, the COVID-19 pandemic impacted healthcare facilities across the country. Along with most outpatient clinics, the EDS Clinic staff were redirected to COVID-19–related activities. As a result of the pandemic, patients were unable to travel if outside of the immediate area. The team implemented virtual visits for the initial evaluation and follow-up appointments to navigate the shift in healthcare. Patient satisfaction varied with virtual visits.

#### Care Model

The EDS Clinic is a tertiary care model rather than a primary care model. The clinical pathways designed were intentional to provide accessibility to as many patients as possible and contribute to the overall body of research. For each patient, the clinical team completed a full diagnostic work-up, assisted the patient in creating a treatment plan, and transitioned the patient to their local primary care provider for ongoing care.

### Discussion

The Mayo Clinic Model of Care is defined by high-quality, compassionate multidisciplinary medical care delivered in an integrated academic institution [22, 23], and formed the foundation for our new multidisciplinary EDS Clinic. This clinic has been successful because of its ability to provide multidisciplinary care consisting of an initial visit to diagnose the condition and establish care and a subsequent visit(s) to identify further modalities for individualized treatment. The direct feedback from the multi-disciplinary team has been positive, by providing a home to patients with a care team that is eager to help them. Interaction with researchers who had characterized the hEDS/HSD population previously seen at Mayo Clinic prior to the start of the EDS Clinic was pivotal to the success of the clinic as well. Their research was used as evidence for the need and feasibility of the Clinic as well as ascertaining key elements to include in the intake questionnaire. Equally important was interacting with patients with hEDS/HSD to better understand the needs of the community and the breadth of the comorbidities associated with the condition to better anticipate referrals. The Clinic works closely with basic, clinical, and translational researchers studying hEDS/HSD.

Ultimately, the Clinic focuses on individualizing care at the patient level. This is particularly important with such a broad and multi-system condition. To this end, treatment for patients includes the primary goal of improving quality of life by focusing on improving physical function with strategies for maintenance with physical and occupational therapists experienced with hypermobility, symptom control and mental wellness.

### Conclusion

Much has been learned through the past on models of patient care. Biomedical research has progressed as well to allow us to treat patients with the tools of medicine we have available. Unfortunately, there is often much that cannot yet be explained by biomedical knowledge, specifically pertaining to the link of illness and disease in patients with hEDS/HSD. Thus, the goal of our medical clinic is to approach each patient with a biopsychosocial understanding. For this reason, to be effective in our treatment — maintaining function and procuring the highest quality of life for our patients, we take a shared responsibility among providers of multiple backgrounds and expertise. We anticipate that this partnership of sharing responsibilities and experiences among healthcare team members will generate improved outcomes, and decrease the time and suffering from onset of symptoms to the diagnosis of hEDS/HSD. As this is a newly established clinic, we look forward to expanding clinical outcome data over time as our treatment progresses.

## Data Availability

Data will be provided upon request via the corresponding author.

## Abbreviations

**EDS**: Ehlers-Danlos syndrome
**hEDS**: Hypermobile Ehlers-Danlos syndrome
**HSD**: Hypermobility spectrum disorder
**VUS**: Variant of unknown significance

## Code availability

N/A.
